# Balancing opportunity and exploitation: unpaid internships in sports nutrition

**DOI:** 10.3389/fnut.2024.1462046

**Published:** 2024-10-23

**Authors:** Nicola Brown

**Affiliations:** Faculty of Sport, Technology and Health Sciences, St Mary's University, Twickenham, United Kingdom

**Keywords:** unpaid internships, sports nutrition, graduate employment, work placements, industry standards, supervision

## Introduction

In recent years, the sports nutrition industry has seen significant growth and investment. With clubs and organizations recognizing the vital role nutrition plays in athletic performance, there has been a surge in opportunities for students to gain hands-on experience in this field. However, not all opportunities are created equal, and the prevalence of unpaid internships and placements raises critical questions about equity and accessibility.

The concept of unpaid internships as a gateway to career advancement in sports nutrition is well-established. Historically, internships in the sporting industry have been viewed as rites of passage, providing crucial industry exposure while students navigate between academic coursework and professional practice (1, 2). However, the reliance on unpaid labor underscores a broader issue: while students seek valuable learning experiences and networking opportunities, sports organizations often exploit their enthusiasm by replacing paid positions with unpaid internships (2, 3). Unpaid internships can undoubtedly offer valuable learning experiences. They provide students with a chance to apply their theoretical knowledge in real-world settings, network with professionals, and enhance their resumes (4–7). However, when these internships require substantial time commitments and responsibilities, the line between opportunity and exploitation becomes blurred.

## Opportunities and exploitation: blurred lines

A recent placement opportunity advertised an unpaid role for a part-time sports nutrition student to assist in delivering nutrition education across various age groups, with a commitment of 21 h per week for a 10-month period. While the experience might be enriching, the lack of financial compensation presents a significant barrier for many students. This is just one example of many “opportunities” to gain valuable experience in a professional sports nutrition environment that are regularly advertised across a number of platforms, or are received by educators to share with their students.

The reality is that not all sports nutrition students (or students in other sports disciplines) have the luxury of working unpaid for 21 h a week. Today's students face mounting financial pressures, including rising tuition fees and living costs (8). They often need to work part-time jobs to support themselves through their studies, limiting their ability to engage in such demanding unpaid internships that do not provide financial compensation and require extensive time commitments (9, 10, 17). This creates an inequitable playing field where only those with sufficient financial backing can afford to take on these roles, excluding talented students who might bring diverse perspectives and innovative ideas to the industry. This is further exacerbated when there is requirement to earn academic credit for internships, particularly during summer terms when students might otherwise be earning income from paid employment (3, 10).

Furthermore, positioning these roles as opportunities for “driven and motivated” students, as often seen in these unpaid placement/internship adverts, can be demeaning, as it assumes that those unable to undertake unpaid internships are somehow less motivated or unwilling to put in the work. In reality, many capable and ambitious students are constrained by circumstances beyond their control, such as financial responsibilities or the need to support their families. This narrative overlooks the systemic barriers that perpetuate inequality in the sports nutrition field. Furthermore, research indicates that being in an unpaid internship role can negatively impact motivation, with interns focusing on seeking other opportunities (11). Whereas paying interns is reported to increase motivation, and satisfaction and facilitate maximum learning (5).

## Learnings from the wider sports industry: limited progress

Research across various sports disciplines highlights persistent issues with unpaid internships, reflecting broader challenges across the sports sector. A survey by the UK Strength and Conditioning Association (UKSCA) in 2016 highlighted that many internships in Strength and Conditioning remain unpaid, leading to discussions about the sustainability and fairness of these practices (12). A report by UK Coaching in 2018 highlighted the prevalence of unpaid coaching roles and their detrimental impact on diversity and accessibility within coaching profession (13). Furthermore, Malone (14), highlighted the challenges faced by sport science students due to unpaid internships, advocating for improved compensation and support structures. In the sports management industry (18) highlighted how unpaid internships create barriers for students from less privileged backgrounds, potentially leading to a less socioeconomically diverse workforce in the sports industry.

These studies collectively highlight that while the sports industry acknowledges the value of practical experience, the persistence of unpaid internships across various disciplines suggests a systemic reluctance to address equity and fairness. Despite ongoing discourse and sporadic research highlighting the inequities and practical difficulties faced by interns, meaningful industry-wide reforms have been slow to materialize. As the demand for practical experience continues to grow, particularly in competitive fields like sports nutrition, it becomes increasingly urgent to re-evaluate and reform internship practices to ensure they are fair, equitable, and supportive of all aspiring professionals.

## Finding a balance: fair opportunities for all

Internships can provide invaluable experience and insights for students, but there needs to be a balance. Some level of unpaid work may be necessary, offering significant learning opportunities through short-term projects, shadowing professionals, or limited hours per week with appropriate supervision. However, expecting students to shoulder significant workloads without compensation is not acceptable. At the very least, expenses should be fully reimbursed, and internships should be structured to ensure that students gain more than they give. This means providing mentorship, educational opportunities, and realistic workloads that can be balanced with other commitments.

It is important to acknowledge that internships embedded within educational frameworks can provide exposure to real-world practices under supervised conditions, serving both educational and experiential purposes. This structured, educational approach differs significantly from internships undertaken post-graduation, where individuals are not gaining academic credit but rather seeking entry into the workforce. When graduates accept unpaid or minimally compensated internships in pursuit of full-time employment, the broader job market is impacted, inadvertently contributing to lower wages across the field by filling critical roles that might otherwise warrant fair compensation. Furthermore, this raises broader concerns about professional standards when critical nutritional support roles are filled by unpaid interns rather than experienced professionals. This can lead to inadequate or inappropriate nutritional guidance for athletes, posing tangible risks to athlete health and performance and undermining the recognition of sports nutrition as a vital, specialized field requiring expert knowledge and experience. Issues such as RED-S, characterized by energy deficiency that impacts physiological function, and related concerns like under-fueling and body image issues, are increasingly recognized as critical in athlete health (15, 16). Additionally, internships and placements often involve supporting youth academy players, adding another level of complexity to the responsibilities entrusted to inexperienced practitioners. Without proper guidance and oversight, inexperienced sports nutrition practitioners may inadvertently contribute to or exacerbate these complex issues among athletes, highlighting the importance of structured mentorship and professional oversight in sports nutrition practices.

## Improving internships and placements: a call to action

To address these challenges and improve the sports nutrition industry, several key strategies can be adopted. While it is acknowledged that these recommendations will not all be implemented overnight, this is just a starting point from which to develop good practice. By taking gradual steps toward these improvements, we can create a more inclusive and equitable environment for future sports nutritionists.

1. **Paid internships:** ensuring that internships and placements offer fair compensation should become the standard. This can be through hourly wages, stipends, or scholarships that cover living expenses. Relevant employment laws and regulations should be followed where applicable.
2. **Expense reimbursement:** at an absolute minimum, internships should fully reimburse students for travel and other related expenses. This can help alleviate the financial burden and make these opportunities accessible to a broader range of students.
3. **Mentorship programs:** strong mentorship structures should be established where experienced professionals guide interns, providing regular feedback and support. This can help ensure that interns are learning and developing their skills effectively.
4. **Balanced workloads:** designing internships with realistic and balanced workloads is crucial. Interns should not be expected to perform the duties of full-time staff members without appropriate support and supervision.
5. **Educational opportunities:** formal training sessions, workshops, and seminars should be offered as part of the internship. This ensures that interns are continuously learning and gaining new skills relevant to their field.
6. **Short-term projects:** integrating short-term placement/internship opportunities that provide valuable experience without requiring long-term unpaid commitments. This allows students to gain experience without the need for substantial unpaid work.
7. **Collaborative efforts:** collaboration between academic institutions and industry partners should be encouraged to create standardized internship programs that are well-regulated and inclusive. This ensures consistency in the quality and structure of internships across the industry.

## Conclusion

The sports nutrition industry must reflect on how it structures internships and placements, or it risks undermining the quality of the professionals they aim to cultivate. Unpaid internships, if not carefully designed, risk perpetuating inequality and limiting the potential contributions of talented and ambitious students. Continued advocacy and collaboration between industry stakeholders and academic institutions are essential to effecting meaningful change. By ensuring opportunities are accessible and fair, we can nurture a diverse cohort of skilled professionals, ensuring that all students, regardless of their financial background, have an equitable chance to impact athlete health and performance positively. Through these efforts, we envision a sports nutrition industry that is both inclusive and dynamic, setting a standard for fairness and excellence in professional practice.
